# Machine Learning-Based Ensemble Classifiers for Anomaly Handling in Smart Home Energy Consumption Data

**DOI:** 10.3390/s22239323

**Published:** 2022-11-30

**Authors:** Purna Prakash Kasaraneni, Yellapragada Venkata Pavan Kumar, Ganesh Lakshmana Kumar Moganti, Ramani Kannan

**Affiliations:** 1 School of Computer Science and Engineering, VIT-AP University, Amaravati 522237, Andhra Pradesh, India; 2 School of Electronics Engineering, VIT-AP University, Amaravati 522237, Andhra Pradesh, India; 3 Department of Electrical and Electronics Engineering, Universiti Teknologi Petronas (UTP), Seri Iskandar 32610, Malaysia

**Keywords:** classification, data anomalies, data imputation, energy consumption data, ensemble classifiers, machine learning, smart home data, smart meter data, tracebase dataset

## Abstract

Addressing data anomalies (e.g., garbage data, outliers, redundant data, and missing data) plays a vital role in performing accurate analytics (billing, forecasting, load profiling, etc.) on smart homes’ energy consumption data. From the literature, it has been identified that the data imputation with machine learning (ML)-based single-classifier approaches are used to address data quality issues. However, these approaches are not effective to address the hidden issues of smart home energy consumption data due to the presence of a variety of anomalies. Hence, this paper proposes ML-based ensemble classifiers using random forest (RF), support vector machine (SVM), decision tree (DT), naive Bayes, K-nearest neighbor, and neural networks to handle all the possible anomalies in smart home energy consumption data. The proposed approach initially identifies all anomalies and removes them, and then imputes this removed/missing information. The entire implementation consists of four parts. Part 1 presents anomaly detection and removal, part 2 presents data imputation, part 3 presents single-classifier approaches, and part 4 presents ensemble classifiers approaches. To assess the classifiers’ performance, various metrics, namely, accuracy, precision, recall/sensitivity, specificity, and F1 score are computed. From these metrics, it is identified that the ensemble classifier “RF+SVM+DT” has shown superior performance over the conventional single classifiers as well the other ensemble classifiers for anomaly handling.

## 1. Introduction

Considering the global thrust towards the development of grid-independent and green energy systems for addressing the unrelenting growth of loads as well as environmental pollution, smart home and renewable energy-based microgrid culture has been increasing worldwide. Smart cities are new-era establishments where all the smart homes are jointly operated to consolidate and optimize electricity utilization. As these establishments are realized with a combination of electrical, communication, and information technology, the gathering of quality data is a challenging task. Smart homes connected to the power network continuously generate huge volumes of energy consumption data, which is normally a combination of timestamps and readings. The reading information in this data is a key value that helps in understanding the energy consumption behavior, billing generation, load profiling, forecasting, contingency analysis, device health condition analysis, etc. All these operations rely upon the quality of the data being captured. However, this data often may consist of different anomalies, viz., garbage data, outliers, redundant data, and missing data due to malfunctioning of advanced metering infrastructure, failure of communication channels, unanticipated issues in power networks, etc. If these anomalies are left unhandled in the dataset, there will be an adverse effect on the system operations and further delude the analytics of the energy consumption data. So, handling these anomalies is highly essential to enable analysts to perform accurate energy data analytics. Thus, the multifaceted nature of the smart home data when compared to other datasets gains importance in the data analysis field. So, this becomes an important research focus for data analysts when compared to the datasets of other applications. This is the prime motivation for the proposed work of this paper.

Big data refers to a huge quantity of data. However, the data quality is a more complex and significant aspect than the quantity in the direction of research [1]. Moreover, the issues related to data quality have gained much importance and attention in energy big data analytics [2]. The increased use of several intelligent devices in power system applications has become the major source of big data, which reflects on data storage, data processing, and data quality [3,4,5]. The failure of these intelligent devices makes data incomplete during the acquisition of energy consumption data. This incomplete data is commonly referred to as missing data [6,7,8]. Handling rather than ignoring this missing data drives toward better data analytics on energy consumption [9]. Hence, it is essential to analyze and impute the missing data in the smart home energy consumption data. Following this, several state-of-the-art works on missing data imputation and ensemble methods are discussed as follows. 

The researchers suggested several ML-based imputation methods as well as thorough benchmarks for the comparison between conventional and modern methods [10]. An imputation algorithm “opt.impute” was introduced in [11] to achieve the finest solutions to the missing data. Further, an extensive review was conducted on the imputation of missing data using ML which helps in understanding the limitations of ML imputation methods [12]. A framework was implemented to improvise the multivariate imputation by chained equations (MICE) in imputing the missing sensor data [13]. A graph-based method was discussed in [14] to impute the missing sensor data. A copy-paste imputation method was introduced in [15] to impute the time-series data of energy. A mixture factor analysis method was discussed to estimate the missing data in the building’s energy load [16]. Different imputation methods, viz., MICE, KNN, and RF-based imputation were implemented to impute the missing data in the sensor data of the internet of things [17]. A data splitting-based imputation method named “nullify the missing values before the imputation” was proposed to impute the missing data [18]. 

A new statistical and ML-based imputation method was implemented in [19] to impute missing data in the applications of power grids. A fuzzy inductive reasoning method was discussed to deal with the missing data during the forecasting process in smart grids [20]. A six-stage particle swarm optimization imputation method was implemented for smart meter data collected from an Indian institution [21]. An imputation method based on a denoising autoencoder was presented in [22]. An imputation model named “bagged averaging of multiple linear regression” was discussed in [23] for imputing missing data in phasor measurement units. A two-stage deep autoencoder-based data imputation method was discussed in [24] for imputing missing data in wind farms. A bagging algorithm was implemented to impute the missing data in time-series data [25]. An autoencoder neural network was presented to impute missing data for classification [26]. The appropriate selection of the best imputation method and classification was discussed in [27]. An extensive study on the packages available in “R” for data imputation was presented in [28]. Electricity theft detection in smart grids using various ML algorithms and deep learning techniques was discussed in [29,30]. An AdaBoost ensemble model was implemented to detect electricity theft [31]. An improvised ensemble model of a general regression neural network and successive geometric transformations model was presented in [32] to recover the partial or fully missed data. 

In summary, the abovementioned literature discusses the concepts of big data, sources of big data, and energy data analytics. In addition, the importance of handling anomalies in big data was discussed. To handle the anomalies in energy consumption datasets, a few imputation methods such as data splitting, fuzzy inductive reasoning, denoising autoencoder, and bagging are used. Further, to evaluate their performance, various single classifiers, namely, SVM, neural networks, etc., are used. However, these approaches are found ineffective to address the hidden issues of smart home energy consumption data due to the presence of a variety of anomalies such as garbage data, outlier data, redundant data, missing data, etc. 

On the other hand, in recent days, the ensemble classification approach is supporting effective classification in data imputation in different applications, which was not tried for the smart home energy consumption data. With this motivation, this paper proposes ML-based ensemble classifiers to handle all the possible anomalies in smart home energy consumption data. The major contributions of this paper are summarized as follows:

▪ The proposed approach initially identifies all anomalies and removes them, and then imputes this information. The entire implementation consists of four parts.
  - Part 1 (anomaly detection and removal) considers the original dataset and refines it by removing all the identified anomalies.
  - Part 2 (data imputation) considers this refined dataset and performs the missing data imputation using median, KNN, and bagging imputation methods, thereby producing an anomaly-free dataset.
  - Part 3 (single-classifier approaches) performs the classification of the dataset using the conventional single-classifier approaches such as RF, SVM, DT, NB, KNN, and NNET.
  - Part 4 (ensemble classifiers approaches) performs the classification of the dataset using the proposed ensemble classifier approaches such as RF+SVM+DT, RF+SVM+NB, RF+SVM+KNN, RF+SVM+NNET, RF+DT+NB, RF+DT+KNN, RF+DT+NNET, RF+NB+KNN, RF+NB+NNET, and RF+KNN+NNET.
▪ To assess the classifiers’ performance, various metrics, namely, accuracy, precision, recall/sensitivity, specificity, and F1 score are computed. From these metrics, it is identified that the ensemble classifier “RF+SVM+DT” has shown superior performance over the conventional single classifiers as well the other ensemble classifiers for anomaly handling in smart home energy consumption data.

All these contributions are structured in the paper as follows. Section 2 presents the description of the dataset. Section 3 presents the description and implementation of the proposed approach. Section 4 presents simulation results and their discussion. Finally, Section 5 concludes the outcomes of the paper in a synopsized way.

## 2. Description of Dataset

To implement the proposed approach, the data of an appliance (refrigerator) from the Tracebase dataset [33] is considered. This dataset consists of 43 different appliances with 158 device IDs that are connected to various smart homes/buildings. Each appliance consists of CSV files that represent the energy consumption data of a day. A detailed description of this dataset can be obtained from [34]. Further, this dataset was considered and used in various literary works. The Tracebase dataset was used in the extensive study of different non-intrusive load monitoring (NILM) power consumption datasets described in [35,36,37]. The present and the future directions for energy management techniques using NILM datasets are discussed in [38]. 

The CSV file (dev_98C08A_2011.09.17.csv) data of the refrigerator appliance is prepared with the columns such as CAPTURED_DATE, CAPTURED_HOUR, CAPTURED_MINUTE, CAPTURED_SECOND, and CAPTURED_READING for implementing the proposed ensemble classifier approach.

## 3. Description and Implementation of the Proposed Approach

**Figure 1:**
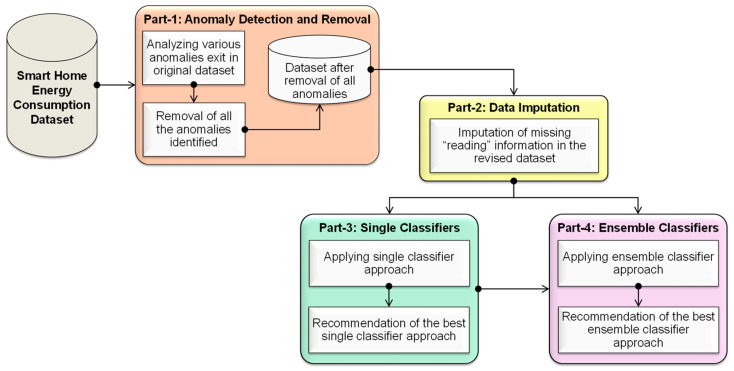
Conceptual model for the proposed approach.

The conceptual model of the proposed approach is shown in Figure 1. It consists of four parts, viz., Part 1, Part 2, Part 3, and Part 4. The smart home energy consumption dataset will be given as input to Part 1. In Part 1, an analysis of the missing data will be carried out for understanding the missingness in the original dataset. Further, the identification and removal of different anomalies (viz., garbage data, outliers in the data, and redundant data) will be performed. From this, a dataset with the abovementioned anomalies removed will be produced and given as input to Part 2. In Part 2, the imputation of missing data will be completed. In Part 3, a single-classifier approach will be applied. This will provide a recommendation of the best single-classifier approach as the output. By taking this best single-classifier as the basis, the ensemble classifiers approach will be applied in Part 4. This will provide a recommendation of the best ensemble classifier to perform the imputation.

**Figure 2:**
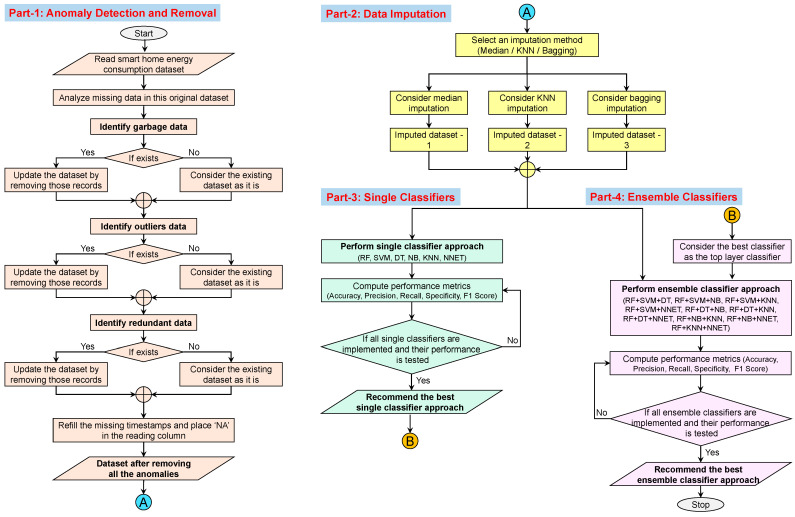
Implementation flow of the proposed approach.

The implementation flow of the proposed ensemble classifiers approach through all the proposed parts is shown in Figure 2. The detailed description and implementation processes are discussed in Section 3.1, Section 3.2, Section 3.3 and Section 3.4 respectively for Part 1, Part 2, Part 3, and Part 4.

### 3.1. Implementation of Part 1 (Anomaly Detection and Removal)

The process starts from Part 1 by reading the smart home energy consumption dataset and saving it in an object “shec_dat”. Initially, the missing data information in this “shec_dat” is analyzed [6]. Further, the process is continued with the identification of garbage data in the dataset. To identify the garbage data i.e., the data other than the numerical data, a function *grepl(“[[:digit:]]* is used on each column of the dataset. If garbage data exists, those records are removed and the remaining data are given as input to identify the outliers data. If there is no garbage data, the existing dataset is used as it is and is given as input to identify the outliers data. The outliers data is the data that does not exist within the expected range. To identify outliers data, a boxplot analysis is applied to the data obtained after removing the garbage data. The boxplot analysis is a standardized approach to showing data distribution in a five-number summary (i.e., minimum, first quartile, median, third quartile, and maximum). The data that lies in between the “minimum” and “maximum” values are considered as the data within the range and useful for the analysis. The data that lies below the “minimum” and above the “maximum” values are considered outliers data and needs to be removed to achieve better analytics. The function boxplot() is applied to the readings column by using *boxplot(shec_dat$CAPTURED_READING, plot=F)$out*. If the outliers exist in the readings column, those records are removed and the remaining data are given as input to identify the redundant data. If there are no outliers, the existing dataset is used as it is to identify the redundant data. In general, redundant data refers to the duplication of the entire record in the dataset. However, in this case, there exist two types of redundant data in the dataset. They are the records with the same timestamp and same reading information, and records with the same timestamp and different reading information. The detailed process of identifying these types of redundant data is discussed in [39]. If the abovementioned types of redundant data exist, those records are removed. If there are no redundant data, the existing data is used as it is to perform the next step. At the end of Part 1, a dataset is obtained after removing all the anomalies (garbage data, outliers data, and redundant data). As several records in the dataset are removed due to the existence of different anomalies, this dataset consists of missing timestamps. Hence, these missing timestamps are filled, and the respective reading information is set to “NA (Not Available)” [8] before proceeding to the implementation of Part 2.

### 3.2. Implementation of Part 2 (Data Imputation)

Once all the missing records are finalized in the dataset obtained after removing all the anomalies, the imputation methods such as median imputation, KNN imputation, and bagging imputation are applied. The implementation of these imputation methods produces datasets with imputed reading values. Further, the single-classifier approach is applied to these imputed datasets. The implementation of the median, KNN, and bagging imputation methods is discussed in Section 3.2.1, Section 3.2.2 and Section 3.2.3 respectively.

#### 3.2.1. Implementation of the Median Imputation Method

In the median imputation method, the median value of the reading information in the CAPTURED_READING column is calculated, and that value is used for imputing the missing reading information. This imputation method is simple and fast. The process of calculating the median value starts with the ordering of readings information in ascending order. Once the ordering of readings information is done, then the number of values (odd or even) in the CAPTURED_READING is taken into consideration. Here, the number of values plays a major role in calculating the median value of the readings information. The formula for calculating the median value is given in Equation (1).

(1) $$Median(D)=\{\begin{array}{cc} D\left(\frac{s+1}{2}\right) & if\;s\;is\;odd\\ \frac{D\left(\frac{s}{2}\right)+D\left(\frac{s}{2}+1\right)}{2} & if\;s\;is\;even \end{array}$$

where *D* = list of values ordered in the CAPTURED_READING column, and *s* = number of values in the CAPTURED_READING column.

If the number of values in the CAPTURED_READING column is odd, then the middle value is considered as the median. If the number of values is even in the CAPTURED_READING column, then the average of the middle two values is considered as the median.

#### 3.2.2. Implementation of the KNN Imputation Method

In the KNN imputation method, the distance between the k-nearest neighbor values is calculated by using the Euclidean distance metric. In the CAPTURED_READING column, the distance between the k-closest samples of the readings is calculated and that distance value is used to impute the missing reading information. The formula for calculating Euclidean distance is given in Equation (2).

(2) $$dist(p,q)=\sqrt{\sum_{i=1}^{m}{\left(p_{i}-q_{i}\right)}^{2}}$$

where *dist* = Euclidean distance, *m* = number of points, and *p_i_* & *q_i_* are the points.

#### 3.2.3. Implementation of the Bagging Imputation Method

In the bagging imputation method, the term ‘bagging’ refers to bootstrap aggregation. The bootstrap is a statistical technique of iteratively resampling the data with replacement in the dataset. To perform this, initially, the number of bootstrap samples is to be fixed, and then the sample size. For each sample of bootstrap the following steps are performed: draw the sample with replacement, fit the model, anticipate the performance of the model based on the out-of-bag sample, and calculate the average of the sample of the model. The multiple iterations of sampling improve the prediction performance of the model. The bagging method fits a bagged tree. This method is simple, powerful, and accurate to impute the missing values in the readings information. However, it is computationally high-cost.

### 3.3. Implementation of Part 3 (Single-Classifier Approach)

**Figure 3:**
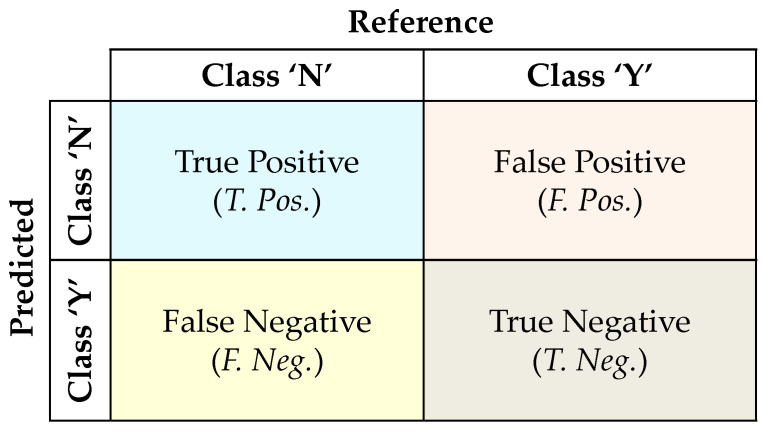
Confusion matrix.

In this section, the single-classifier approach is performed using various classifiers, viz., RF, SVM, DT, NB, KNN, and NNET, for the classification. All these classifiers are implemented individually on the dataset. To implement these, the dataset is divided into train_set and test_set. These classifiers are trained on the train_set using k-fold cross-validation. Here, the k-value considered is 10. Further, these classifiers are applied to the test_set to predict the classes Yes (Y) or No (N). Here, class ‘Y’ represents missing data, and class ‘N’ represents non-missing data. After the implementation, the performance metrics such as accuracy, precision, recall/sensitivity, specificity, and F1 score are computed using a confusion matrix to evaluate each classifier’s performance. The confusion matrix is shown in Figure 3 and the formulae for computing the performance metrics are given in Equations (3)–(7).

(3) $$Accuracy=\frac{T.Pos.+T.Neg.}{T.Pos.+T.Neg.+F.Pos.+F.Neg.}$$

(4) $$Precision=\frac{T.Pos.}{T.Pos.+F.Pos.}$$

(5) $$Recall/Sensitivity=\frac{T.Pos.}{T.Pos.+F.Neg.}$$

(6) $$Specificity=\frac{T.Neg.}{T.Neg.+F.Pos.}$$

(7) $$F1Score=\frac{2*(Precision*Recall)}{\left(Precision+Recall\right)}$$

If all the single classifiers are implemented and their performance is verified, then, the best single classifier is recommended. Otherwise, the performance metrics are re-verified.

### 3.4. Implementation of Part 4 (Ensemble Classifiers Approach)

**Figure 4:**
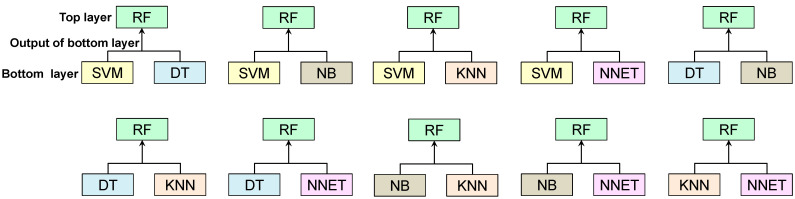
Stacking of classifiers.

This section uses the best single classifier recommended in Part3 as the input to develop ensemble classifiers. The ensemble of classifiers is performed using the “stacking” method. In stacking, there are two layers called the top layer and the bottom layer. The top layer consists of a classifier, which is referred to as a base classifier and the bottom layer consists of other classifiers. The output of the bottom layer is given as input to the top layer. The classifier used in the top layer is an ensemble with the output of the bottom layer classifiers, which produces an ensemble classifier. The stacking of classifiers is shown in Figure 4. From this figure, it is seen that the single classifiers used in the bottom layer are an ensemble with the recommended best classifier used in the top layer. For example, the single classifiers SVM and DT are part of the ensemble with the recommended best classifier RF. Similarly, all the other single classifiers form an ensemble with RF and produce ensemble classifiers. To implement these ensemble classifiers, the imputed datasets are given as input. Further, each imputed dataset is divided into train_set and test_set. The ensemble classifiers are trained on the train_set using k-fold cross-validation. Here, the k-value considered is 10. Further, these ensemble classifiers are applied to test_set to predict the classes Y or N. After the implementation, the performance metrics such as accuracy, precision, recall/sensitivity, specificity, and F1 score are computed using a confusion matrix to evaluate each ensemble classifier’s performance. If all the ensemble classifiers are implemented and their performance is verified then the best ensemble classifier for the imputation is recommended, otherwise the performance metrics are re-verified.

## 4. Simulation Results and Discussion

In keeping with the aims of the paper, the simulation results of the implementation are presented in three subsections. Section 4.1, Section 4.2 and Section 4.3 present the results corresponding to anomaly detection and removal, single-classifier approach, and ensemble classifiers approach, respectively.

### 4.1. Results Corresponding to Anomaly Detection and Removal

**Figure 5:**
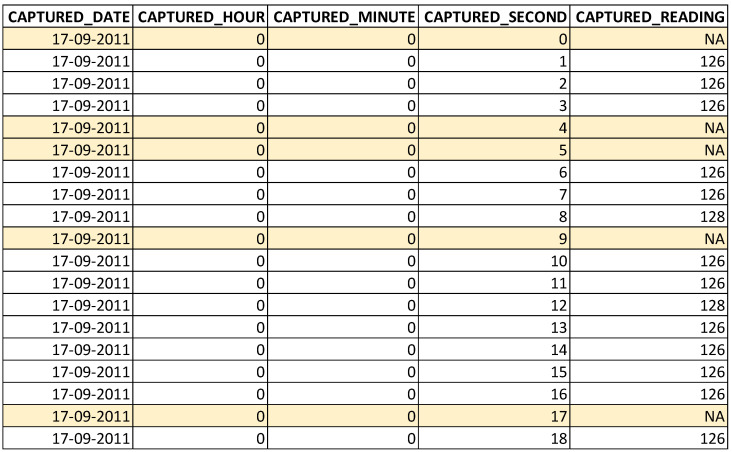
Data after filling missing timestamps and placing NA.

This section presents the details of the missing data in the original CSV file (original dataset) and the missing data in the dataset after eliminating the anomalies. The number of records in this original dataset is 155,374. During the analysis of missing data, 700 records are missed in the original dataset [7]. During the identification of garbage data, no garbage data (other than numerical data) are identified in the original CSV file. Hence, no records are removed and the same number of records (155,374) are available. During the identification of outliers data, there are 25 readings identified as outliers and the respective records are removed from the dataset. The removal of records with outliers left the dataset with 155,349 records. During the identification of redundant data, the records with the same timestamp and same reading are identified and those records are removed from the dataset. This removal left the dataset with 98,779 records. Further, the records with the same timestamp and different readings are identified and those records are removed from the dataset. This removal left the dataset with 72,597 records. Once the redundant data are removed, the missing data are filled with the respective timestamps and the respective reading with NA value, as shown in Figure 5 (all the highlighted rows). After this filling, there are 86,400 records in the dataset, out of these, 13,803 records contain missing readings.

**Figure 6:**
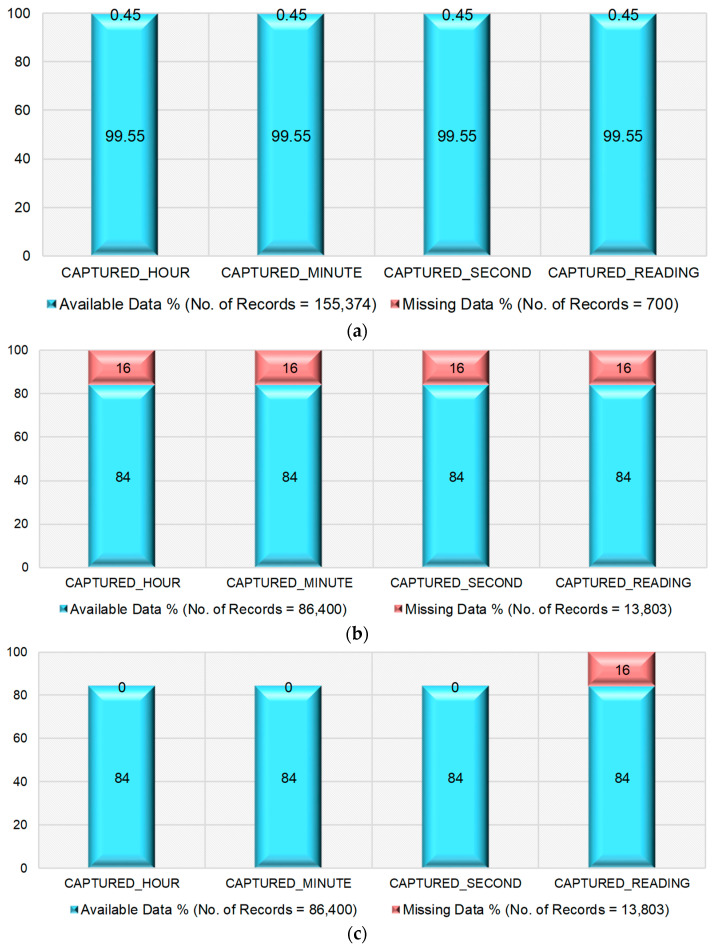
Proportion of missing data in the dataset. (**a**) Original dataset. (**b**) After removing the anomalies. (**c**) After filling missing timestamps.

The proportions of the available data and missing data in the original dataset and the dataset available after removing anomalies are shown in Figure 6a–c. These figures show the proportion of the missing data and available data in the considered dataset in three different scenarios, namely, (i) consideration of the original dataset, (ii) consideration of the dataset that is obtained after removing the anomalies, and (iii) consideration of the dataset after filling the missing timestamps, ready for the imputation.

From Figure 6a, it is understood that the proportion of available data is 99.55% and missing data is 0.45% in the original dataset. From Figure 6b, it is seen that the proportion of available data is 84% and missing data is 16% in all columns of the dataset obtained after removing anomalies. From Figure 6c, it is evident that there are no missing data in the columns CAPTURED_DATE, CAPTURED_HOUR, CAPTURED_MINUTE, CAPTURED_SECOND and the proportion of data availability is 84%. Further, there are missing readings in the column CAPTURED_READING with a proportion of 16%.

### 4.2. Results Corresponding to the Single-Classifier Approach

This section presents the performance of a single-classifier approach on the imputed datasets. The performance of classifiers in the median, KNN, and bagging imputation methods are discussed in Section 4.2.1, Section 4.2.2 and Section 4.2.3, respectively.

#### 4.2.1. Performance of the Single-Classifier Approach in the Median Imputation Method

**Figure 7:**
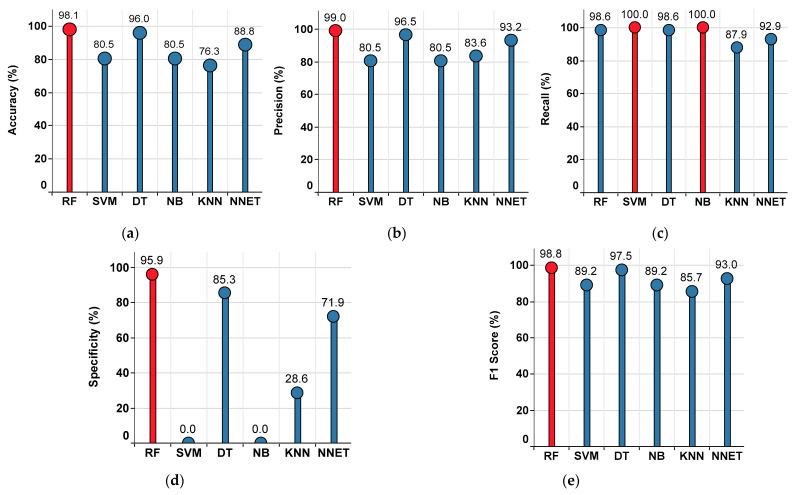
Performance metrics for the single-classifier approach on the median imputed dataset. (**a**) Accuracy (**b**) Precision. (**c**) Recall. (**d**) Specificity. (**e**) F1 Score.

The performance metrics of each classifier are shown in Figure 7, where the red colored bar(s) indicate the highest value achieved corresponding to that particular metric. From this, the highest accuracy value of 98.1% is observed in RF, while the lowest accuracy value of 76.3% is observed in KNN, as shown in Figure 7a. The highest precision value of 99% is observed in RF, while the lowest precision value of 80.5% is observed in SVM and NB, as shown in Figure 7b. The highest recall value of 100% is observed in SVM and NB, while the lowest recall value of 87.9% is observed in KNN, as shown in Figure 7c. The highest specificity value of 95.9% is observed in RF, while the lowest specificity value of 0% is observed in SVM and NB, as shown in Figure 7d. The highest F1 Score value of 98.8% is observed in RF, while the lowest F1 Score value of 85.7% is observed in KNN, as shown in Figure 7e. From the subplots in Figure 7a–e, it is understood that the classifier RF has outperformed the others. Further, the performance summary of all the single classifiers is given in Table 1.

#### 4.2.2. Performance of the Single-Classifier Approach in the KNN Imputation Method

**Figure 8:**
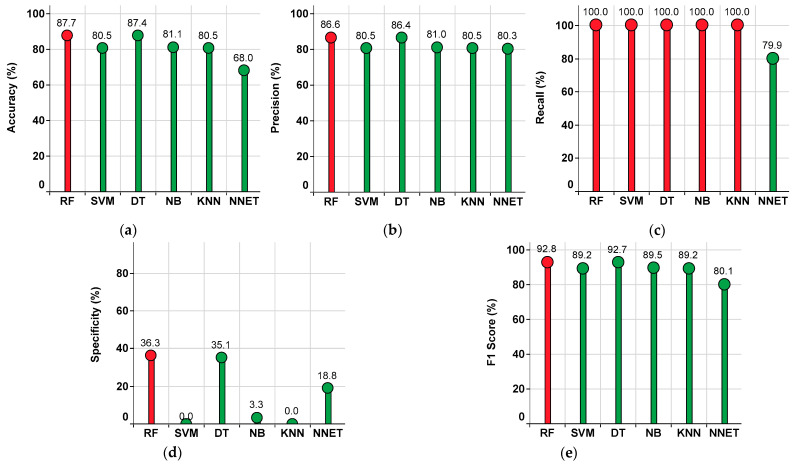
Performance metrics for the single-classifier approach on the KNN imputed dataset. (**a**) Accuracy. (**b**) Precision. (**c**) Recall. (**d**) Specificity. (**e**) F1 Score.

The performance metrics of each classifier are shown in Figure 8, where the red colored bar(s) indicate the highest value achieved corresponding to that particular metric. From this, the highest accuracy value of 87.7% is observed in RF, while the lowest accuracy value of 68% is observed in NNET, as shown in Figure 8a. The highest precision value of 86.6% is observed in RF, while the lowest precision value of 80.3% is observed in NNET, as shown in Figure 8b. The highest recall value of 100% is observed in RF, SVM, DT, NB, and KNN, while the lowest recall value of 79.9% is observed in NNET, as shown in Figure 8c. The highest specificity value of 36.3% is observed in RF, while the lowest specificity value of 0% is observed in SVM and KNN, as shown in Figure 8d. The highest F1 Score value of 92.8% is observed in RF, while the lowest F1 Score value of 80.1% is observed in NNET, as shown in Figure 8e. From the subplots in Figure 8a–e, it is understood that the classifier RF has outperformed the others. Further, the percentage summary of all classifiers is given in Table 2.

#### 4.2.3. Performance of the Single-Classifier Approach in the Bagging Imputation Method

**Figure 9:**
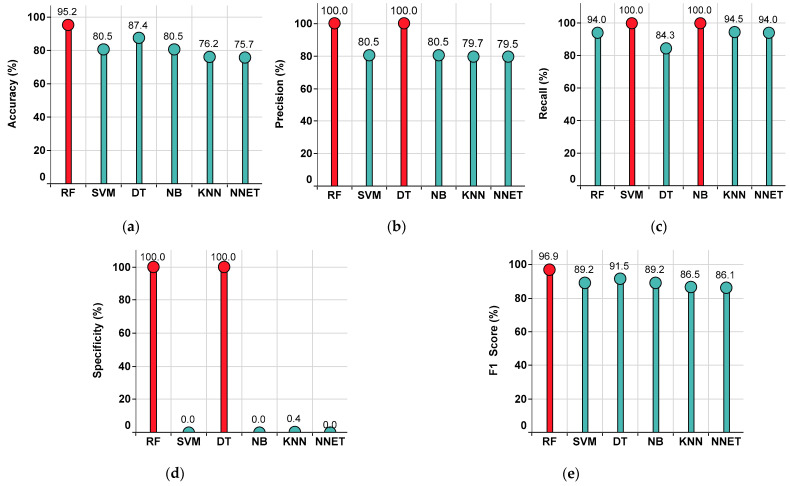
Performance metrics for the single-classifier approach on the bagging imputed dataset. (**a**) Accuracy. (**b**) Precision. (**c**) Recall. (**d**) Specificity. (**e**) F1 Score.

The performance metrics of each classifier are shown in Figure 9, where the red colored bar(s) indicate the highest value achieved corresponding to that particular metric. From this, the highest accuracy value of 95.2% is observed in RF, while the lowest accuracy value of 75.7% is observed in NNET, as shown in Figure 9a. The highest precision value of 100% is observed in RF and DT, while the lowest precision value of 79.5% is observed in NNET, as shown in Figure 9b. The highest recall value of 100% is observed in SVM, and NB, while the lowest recall value of 84.3% is observed in DT, as shown in Figure 9c. The highest specificity value of 100% is observed in RF and DT, while the lowest specificity value of 0% is observed in SVM, NB, and NNET, as shown in Figure 9d. The highest F1 Score value of 96.9% is observed in RF, while the lowest F1 Score value 86.1% is observed in NNET, as shown in Figure 9e. From the subplots Figure 9a–e, it is understood that the classifier RF has outperformed the others. Further, the percentage summary of all classifiers is given in Table 3.

### 4.3. Results Corresponding to the Ensemble Classifiers Approach

This section presents the performance of the ensemble classifiers approaches on the imputed datasets. The performance of ensemble classifiers in the median, KNN, and bagging imputation methods are discussed in Section 4.3.1, Section 4.3.2 and Section 4.3.3 respectively. 

#### 4.3.1. Performance of the Ensemble Classifiers Approach in the Median Imputation Method

**Figure 10:**
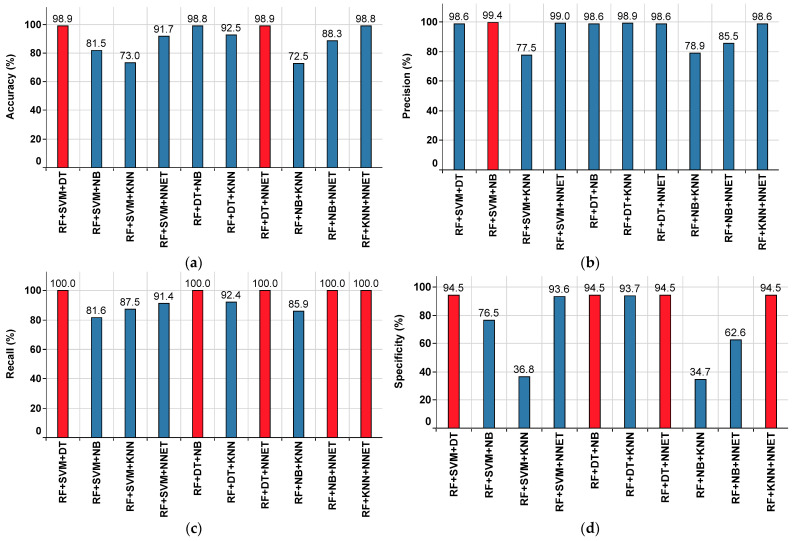

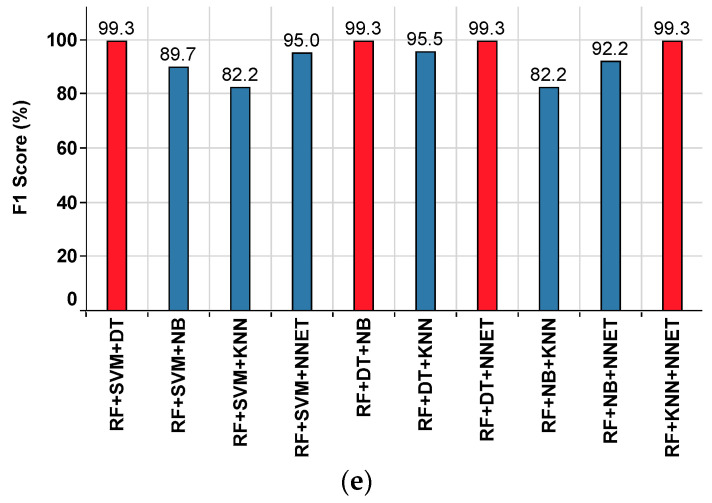
Performance metrics for the ensemble classifiers approach on the median imputed dataset. (**a**) Accuracy. (**b**) Precision. (**c**) Recall. (**d**) Specificity. (**e**) F1 Score.

The performance metrics of each ensemble classifier are shown in Figure 10, where the red colored bar(s) indicate the highest value achieved corresponding to that particular metric. From this, the highest accuracy value of 98.9% is observed in RF+SVM+DT and RF+DT+NNET, while the lowest accuracy value of 72.5% is observed in RF+NB+KNN, as shown in Figure 10a. The highest precision value of 99.4% is observed in RF+SVM+NB, while the lowest precision value of 77.5% is observed in RF+SVM+KNN, as shown in Figure 10b.

The highest recall value of 100% is observed in RF+SVM+DT, RF+DT+NB, RF+DT+NNET, RF+NB+NNET, and RF+KNN+NNET, while the lowest recall value of 81.6% is observed in RF+SVM+NB, as shown in Figure 10c. The highest specificity value of 94.5% is observed in RF+SVM+DT, RF+DT+NB, RF+DT+NNET, and RF+KNN+NNET, while the lowest specificity value of 34.7% is observed in RF+NB+KNN, as shown in Figure 10d. 

The highest F1 Score value of 99.3% is observed in RF+SVM+DT, RF+DT+NB, RF+DT+NNET, and RF+KNN+NNET, while the lowest F1 Score value of 82.2% is observed in RF+SVM+KNN and RF+NB+KNN, as shown in Figure 10e. From the subplots in Figure 10a–e, it is understood that the ensemble classifiers RF+SVM+DT and RF+DT+NNET have outperformed the others. 

Further, the performance summary of all ensemble classifiers with respect to various parameters is given in Table 4.

#### 4.3.2. Performance of the Ensemble Classifiers Approach in the KNN Imputation Method

**Figure 11:**
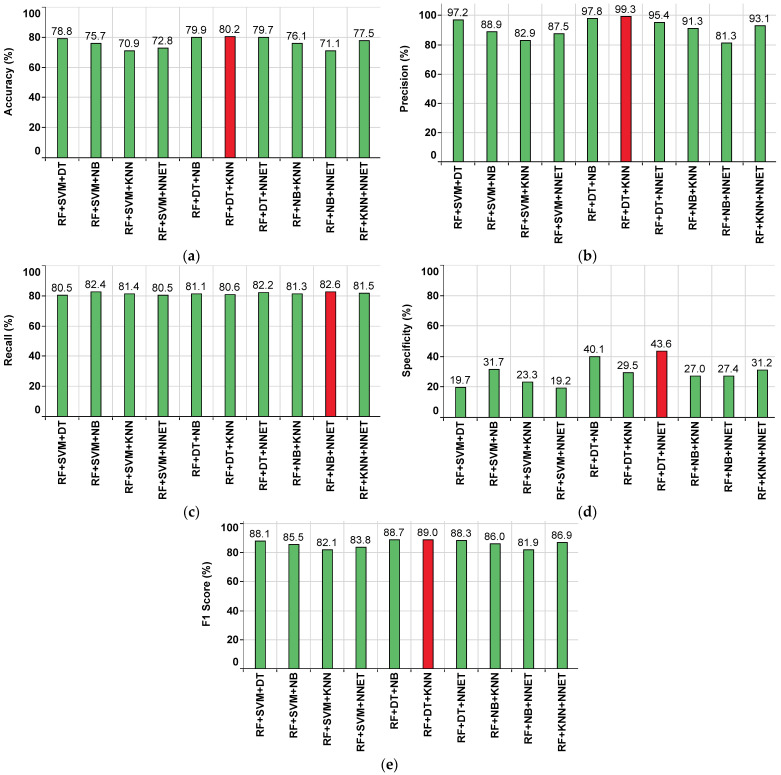
Performance metrics for the ensemble classifiers approach on the KNN imputed dataset. (**a**) Accuracy. (**b**) Precision. (**c**) Recall. (**d**) Specificity. (**e**) F1 Score.

The performance metrics of each ensemble classifier are shown in Figure 11, where the red colored bar(s) indicate the highest value achieved corresponding to that particular metric. From this, the highest accuracy value of 80.2% is observed in RF+DT+KNN, while the lowest accuracy value of 70.9% is observed in RF+SVM+KNN, as shown in Figure 11a. The highest precision value of 99.3% is observed in RF+DT+KNN, while the lowest precision value of 81.3% is observed in RF+NB+NNET, as shown in Figure 11b. 

The highest recall value of 82.6% is observed in RF+NB+NNET, while the lowest recall value of 80.5% is observed in RF+SVM+DT, RF+SVM+NNET, as shown in Figure 11c. The highest specificity value of 43.6% is observed in RF+DT+NNET, while the lowest specificity value of 19.2% is observed in RF+SVM+NNET, as shown in Figure 11d. The highest F1 Score value of 89% is observed in RF+DT+KNN, while the lowest F1 Score value of 81.9% is observed in RF+NB+NNET, as shown in Figure 11e. From the subplots in Figure 11a–e, it is understood that the ensemble classifier RF+DT+KNN has outperformed the others. Further, the performance summary of all ensemble classifiers with respect to various parameters is given in Table 5.

#### 4.3.3. Performance of the Ensemble Classifiers Approach in the Bagging Imputation Method

**Figure 12:**
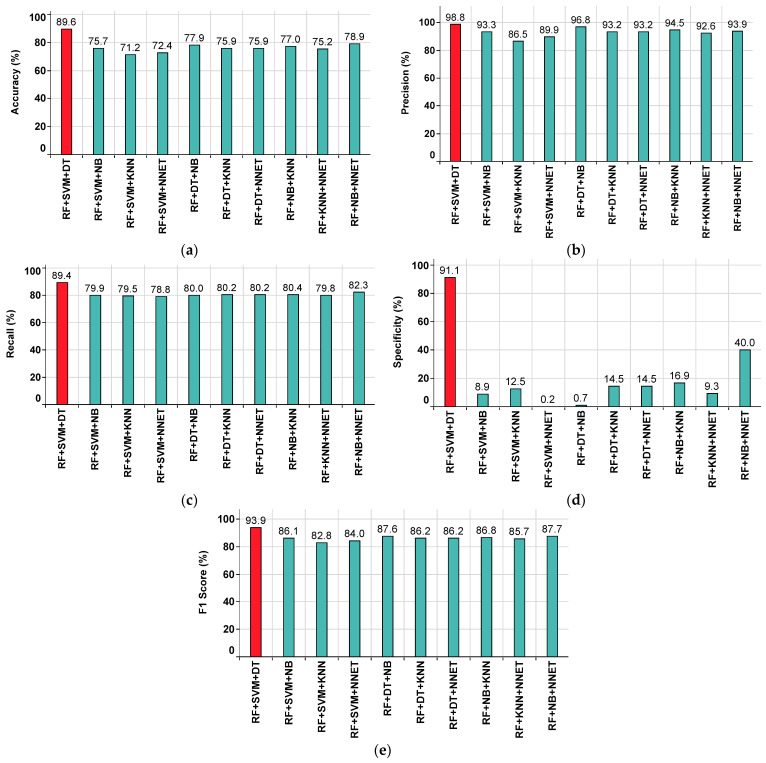
Performance metrics for ensemble classifiers approach on bagging imputed dataset. (**a**) Accuracy. (**b**) Precision. (**c**) Recall. (**d**) Specificity. (**e**) F1 Score.

The performance metrics of each ensemble classifier are shown in Figure 12, where the red colored bar(s) indicate the highest value achieved corresponding to that particular metric. From this, the highest accuracy value of 89.6% is observed in RF+SVM+DT, while the lowest accuracy value of 71.2% is observed in RF+SVM+KNN, as shown in Figure 12a. The highest precision value of 98.8% is observed in RF+SVM+DT, while the lowest precision value of 86.5% is observed in RF+SVM+KNN, as shown in Figure 12b. The highest recall value of 89.4% is observed in RF+SVM+DT, while the lowest recall value of 78.8% is observed in RF+SVM+NNET, as shown in Figure 12c.

The highest specificity value of 91.1% is observed in RF+SVM+DT, while the lowest specificity value of 0.2% is observed in RF+SVM+NNET, as shown in Figure 12d. The highest F1 Score value of 93.9% is observed in RF+SVM+DT, while the lowest F1 Score value 82.8% is observed in RF+SVM+KNN, as shown in Figure 12e. From the subplots in Figure 12a–e, it is understood that the ensemble classifier RF+SVM+DT has outperformed the others. 

Further, the performance summary of all ensemble classifiers with respect to various parameters is given in Table 6.

## 5. Conclusions

This paper proposes a machine learning-based ensemble classifiers approach to address the anomalies present in smart homes’ energy consumption data. This proposed approach has proven to be more effective than the conventional single-classifier approach that is presented in the literature. The salient observations from this work are summarized as follows:

▪ All the possible anomalies are successfully identified and removed from the dataset. The number of records in the original dataset is 155,374 and the number of records available in the refined dataset after removing anomalies is 86,400, which is the actual expected number of records as per the dataset description.
▪ Out of 86,400 records, 13,803 records are identified as records with missing data. This missing data has been successfully imputed by using various imputation methods (median, KNN, and bagging).
▪ To assess the process of imputation, various conventional single-classifier approaches, as well as the proposed ensemble classifiers approaches, are implemented. From the computation of the performance metrics (accuracy, precision, recall/sensitivity, specificity, and F1 score), the RF classifier is identified as the superior single-classifier to all other single classifiers.
▪ Out of the proposed ensemble classifiers, “RF+SVM+DT” has shown superior performance over the conventionally best single classifier (RF) as well the other ensemble classifiers for imputing the missing reading information.

Thus, the proposed ensemble classifiers approach has successfully handled anomalies that exist in the smart home energy consumption data.

### Impacts and Implications of the Work

The proposed work in this paper helps in data preprocessing by the cleansing of data, which is typically essential to carry out precise analytics, and thereby, take superior decisions for energy management in smart buildings. Furthermore, the outcome of this work helps as a ready reference to understand the irregularities of the live data captured in a smart building/home/grid application for better data analytics. This impacts one of the important objectives of “United Nations Sustainable Development Goals (UN SDGs)—SDG 7: Energy” in producing an anomaly-free dataset for providing several customer services.

In addition, the identification of different data anomalies, viz., missing data, outliers data, garbage data, and redundant data in the energy consumption dataset, may be applied to the malfunctioning of metering infrastructure, failure/glitches of communication channels, cyber-attacks, energy thefts, unanticipated situations in power networks, etc.

## Figures and Tables

**Table 1:** Performance comparison of single-classifier approach on the median imputed dataset.

Classifier	Accuracy (%)	Precision (%)	Recall (%)	Specificity (%)	F1 Score (%)
RF	98.1	99	98.6	95.9	98.8
SVM	80.5	80.5	100	0	89.2
DT	96	96.5	98.6	85.3	97.5
NB	80.5	80.5	100	0	89.2
KNN	76.3	83.6	87.9	28.6	85.7
NNET	88.8	93.2	92.9	71.9	93
Superior Classifier	RF	RF	SVM, NB	RF	RF

**Table 2:** Performance comparison of the single-classifier approach on the KNN imputed dataset.

Classifier	Accuracy (%)	Precision (%)	Recall (%)	Specificity (%)	F1 Score (%)
RF	87.7	86.6	100	36.3	92.8
SVM	80.5	80.5	100	0	89.2
DT	87.4	86.4	100	35.1	92.7
NB	81.1	81	100	3.3	89.5
KNN	80.5	80.5	100	0	89.2
NNET	68	80.3	79.9	18.8	80.1
Superior Classifier	RF	RF	All except NNET	RF	RF

**Table 3:** Performance comparison of the single-classifier approach on the bagging imputed dataset.

Classifier	Accuracy (%)	Precision (%)	Recall (%)	Specificity (%)	F1 Score (%)
RF	95.2	100	94	100	96.9
SVM	80.5	80.5	100	0	89.2
DT	87.4	100	84.3	100	91.5
NB	80.5	80.5	100	0	89.2
KNN	76.2	79.7	94.5	0.4	86.5
NNET	75.7	79.5	94	0	86.1
Superior Classifier	RF	RF, DT	SVM, NB	RF, DT	RF

**Table 4:** Performance comparison of the ensemble classifiers approach on the median imputed dataset.

Classifier	Accuracy (%)	Precision (%)	Recall (%)	Specificity (%)	F1 Score (%)
RF+SVM+DT	98.9	98.6	100	94.5	99.3
RF+SVM+NB	81.5	99.4	81.6	76.5	89.7
RF+SVM+KNN	73	77.5	87.5	36.8	82.2
RF+SVM+NNET	91.7	99	91.4	93.6	95
RF+DT+NB	98.8	98.6	100	94.5	99.3
RF+DT+KNN	92.5	98.9	92.4	93.7	95.5
RF+DT+NNET	98.9	98.6	100	94.5	99.3
RF+NB+KNN	72.5	78.9	85.9	34.7	82.2
RF+NB+NNET	88.3	85.5	100	62.6	92.2
RF+KNN+NNET	98.8	98.6	100	94.5	99.3
Superior Classifier	RF+SVM+DT RF+DT+NNET	RF+SVM+NB	RF+SVM+DT RF+DT+NB RF+DT+NNET RF+NB+NNET RF+KNN+NNET	RF+SVM+DT RF+DT+NB RF+DT+NNET RF+KNN+NNET	RF+SVM+DT RF+DT+NB RF+DT+NNET RF+KNN+NNET

**Table 5:** Performance comparison of the ensemble classifiers approaches on the KNN imputed dataset.

Classifier	Accuracy (%)	Precision (%)	Recall (%)	Specificity (%)	F1 Score (%)
RF+SVM+DT	78.8	97.2	80.5	19.7	88.1
RF+SVM+NB	75.7	88.9	82.4	31.7	85.5
RF+SVM+KNN	70.9	82.9	81.4	23.3	82.1
RF+SVM+NNET	72.8	87.5	80.5	19.2	83.8
RF+DT+NB	79.9	97.8	81.1	40.1	88.7
RF+DT+KNN	80.2	99.3	80.6	29.5	89
RF+DT+NNET	79.7	95.4	82.2	43.6	88.3
RF+NB+KNN	76.1	91.3	81.3	27	86
RF+NB+NNET	71.1	81.3	82.6	27.4	81.9
RF+KNN+NNET	77.5	93.1	81.5	31.2	86.9
Superior Classifier	RF+DT+KNN	RF+DT+KNN	RF+NB+NNET	RF+DT+NNET	RF+DT+KNN

**Table 6:** Performance comparison of the ensemble classifiers approaches on the bagging imputed dataset.

Classifier	Accuracy (%)	Precision (%)	Recall (%)	Specificity (%)	F1 Score (%)
RF+SVM+DT	89.6	98.8	89.4	91.1	93.9
RF+SVM+NB	75.7	93.3	79.9	8.9	86.1
RF+SVM+KNN	71.2	86.5	79.5	12.5	82.8
RF+SVM+NNET	72.4	89.9	78.8	0.2	84
RF+DT+NB	77.9	96.8	80	0.7	87.6
RF+DT+KNN	75.9	93.2	80.2	14.5	86.2
RF+DT+NNET	75.9	93.2	80.2	14.5	86.2
RF+NB+KNN	77	94.5	80.4	16.9	86.8
RF+NB+NNET	78.9	93.9	82.3	40	87.7
RF+KNN+NNET	75.2	92.6	79.8	9.3	85.7
Superior Classifier	RF+SVM+DT	RF+SVM+DT	RF+SVM+DT	RF+SVM+DT	RF+SVM+DT

## Data Availability

Not applicable.

## Abbreviations

CSV	comma-separated values
DT	decision tree
KNN	K-nearest neighbour
ML	machine learning
MICE	multivariate imputation by chained equations
NA	not available
NB	naive Bayes
NILM	non-intrusive load monitoring
NNET	neural networks
RF	random forest
SVM	support vector machine
